# Comparative Analysis of Model-Based Predictive Shared Control for Delayed Operation in Object Reaching and Recognition Tasks With Tactile Sensing

**DOI:** 10.3389/frobt.2021.730946

**Published:** 2021-09-27

**Authors:** Leone Costi, Luca Scimeca, Perla Maiolino, Thilina Dulantha Lalitharatne, Thrishantha Nanayakkara, Ryman Hashem, Fumiya Iida

**Affiliations:** ^1^ Bio Inspired Robotics Laboratory, Department of Engineering, University of Cambridge, Cambridge, United Kingdom; ^2^ NAVER AI Lab, NAVER Corp, Seongnam-si, South Korea; ^3^ Oxford Robotics Institute, University of Oxford, Oxford, United Kingdom; ^4^ Dyson School of Design Engineering, Imperial College London, London, United Kingdom

**Keywords:** shared control, teleoperation, communication delay, bayesian predictor, human-robot collaboration

## Abstract

Communication delay represents a fundamental challenge in telerobotics: on one hand, it compromises the stability of teleoperated robots, on the other hand, it decreases the user’s awareness of the designated task. In scientific literature, such a problem has been addressed both with statistical models and neural networks (NN) to perform sensor prediction, while keeping the user in full control of the robot’s motion. We propose shared control as a tool to compensate and mitigate the effects of communication delay. Shared control has been proven to enhance precision and speed in reaching and manipulation tasks, especially in the medical and surgical fields. We analyse the effects of added delay and propose a unilateral teleoperated leader-follower architecture that both implements a predictive system and shared control, in a 1-dimensional reaching and recognition task with haptic sensing. We propose four different control modalities of increasing autonomy: *non-predictive human control (HC)*, *predictive human control (PHC)*, *(shared) predictive human-robot control (PHRC)*, and *predictive robot control (PRC)*. When analyzing how the added delay affects the subjects’ performance, the results show that the *HC* is very sensitive to the delay: users are not able to stop at the desired position and trajectories exhibit wide oscillations. The degree of autonomy introduced is shown to be effective in decreasing the total time requested to accomplish the task. Furthermore, we provide a deep analysis of environmental interaction forces and performed trajectories. Overall, the shared control modality, *PHRC*, represents a good trade-off, having peak performance in accuracy and task time, a good reaching speed, and a moderate contact with the object of interest.

## 1 Introduction

Communication delay has been one of the biggest challenges in the field of teleoperated systems, from healthcare applications to construction robotics (Tao et al., 2020; Ferrell, 2013; Anderson and Spong, 1989; Huang et al., 2017) and still represents one of the biggest obstacles that prevent teleoperation from being commonly used in delicate environments, such as healthcare (Mehrdad et al., 2021; Avgousti et al., 2016; Buvik et al., 2016). This can either occur directly in the automatic controller as can be the case for bilateral teleoperation with force feedback, or indirectly by the human operator overcompensating for perceived errors due to delayed responses, giving rise to operator-induced oscillations (Smith and Jensfelt, 2010). In the past, the application of statistical methods and neural networks (NN) (Farajiparvar et al., 2020) have shown promise to solve such a challenge, and simultaneous application of both to multiple leader-follower communication channels has also been explored (Sirouspour, 2005); Liu, 2015; Farahmandrad et al., 2020). Statistical methods are characterized by a clear internal model but have some drawbacks regarding the maximum magnitude of the predictable delay, and the detection of new trajectories’ onset (Takagi et al., 2020; Sharifi et al., 2017). On the other hand, NN-based prediction systems are more versatile, but the interpretability of their “black box” internal models is an issue, and they typically need large amounts of data for training (Huang et al., 2000; Nikpour et al., 2020). The aforementioned delay problem is even more important when we consider haptic feedback, because of the technical challenges raised by the encoding and transmission of such information (Van Den Berg et al., 2017), and of the more complex stabilization of force-based control (Kanaishi et al., 2020; Park and Khatib, 2006; Okamura, 2004). Overall, both strategies result in a delay-free prediction that is shown with or instead of the delayed signal. Depending on the accuracy of the prediction, the estimated signal can contain significant errors that are going to undermine the user’s trust in the prediction. Since the explored strategies exploit systems with 0 degree of autonomy (DoA), the contribution of the delay-free prediction loses effectiveness when it is not being trusted.

In this paper, we focus on shared control as an effective way to overcome the problem of delay. This is achieved by combining a simple predictive system with shared control. The scientific interest for human-robot cooperation and shared control has been exponentially growing in the last 20 years (Ajoudani et al., 2018), especially with regards to teleoperated systems (Luo et al., 2020), and it has been shown to be a suitable substitute to human-human cooperation at times (Ivanova et al., 2020). Human-robot cooperation has been investigated for the achievement of both known and unknown goals (Javdani et al., 2018; Hauser, 2013). When the goal is unknown, the operator’s intention is predicted by using either statistical methods (Tanwani and Calinon, 2017) or neural networks (Reddy et al., 2018). The introduction to some DoA has been proven beneficial to achieve a better performance in a variety of tasks, from reaching motions to track following, and faster task execution times: prior studies show that more autonomy often brings more accuracy and precision (Muelling et al., 2017). Given such an increase in performance, shared control has also been introduced in surgical robotics to achieve higher precision and faster timings (Ficuciello et al., 2019; Attanasio et al., 2021; Abdelaal et al., 2020; Moustris et al., 2011). Shared control has also been shown to help to minimize the effect of sensor noise (Schultz et al., 2017), and has been partially investigated as an answer to compensate delay-induced motions (Inoue et al,. 2009).

Our goal is to study the effect of communication delay and investigate the effect of increasing autonomy as a possible countermeasure in a unilateral teleoperation system. To do so, we implement a discrimination task in which the subjects are asked to remotely control a robot to touch two different objects, one hard and one soft, and correctly identify them via visualization of haptic data. The task will be repeated introducing delay up to 4 s and testing four modalities of increasing autonomy, i.e.: *non-predictive human control (HC)*, *predictive human control (PHC)*, *(shared) predictive human-robot control (PHRC)* and *predictive robot control (PRC)*. This task was chosen as a trade-off between ease of learning by the subjects (which has led to shorter familiarization times with the system), and complexity of haptic interactions, to infer the effect of the increasing DoA as a mitigation tool for the indirect instabilities caused by adding communication delay. In *HC*, the subject can only rely on the delayed data, whereas in *PHC*, the leader develops and updates an internal model as the task is played, and uses such a model to predict real-time sensors’ output. In *PRC* mode, the robot uses the same internal model to select the best position for a correct answer and the subject can only make the decision, but cannot move the robot. Finally, in *PHRC*, the target position selected by the robot and the remote commands are combined and weighted depending on the internal model’s accuracy around the target position.

Our hypothesis is that increasing the autonomy will increase the performance, mainly assessed with accuracy and task time, because it would compensate for the indirect instability induced by the delay. At the same time, we wish to investigate whether shared control can outperform pure robot autonomy or human control, because fully automated trajectories could largely differ from human strategy, therefore frustrating the subject and not achieving ideal cooperation between human and robot. We aim to show the following: *HC* is sensitive to delays, whereas the introduction of the internal model in the other modalities aids delayed control, and the introduction of autonomy (*PHRC* and *PRC*) can improve performance (accuracy, task time, and interaction forces).

In the remainder of the paper, we will introduce the experimental setup in Section 2.1, followed by a detailed description of the different components in the architecture: the leader’s and follower’s control algorithms in Section 2.2.1 and Section 2.2.2, respectively. Finally, Section 3 will show the results obtained in the experimental trials and will be followed by the discussion and closing remarks in Section 4.

## 2 Materials and Methods

### 2.1 Experimental Setup

**FIGURE 1:**
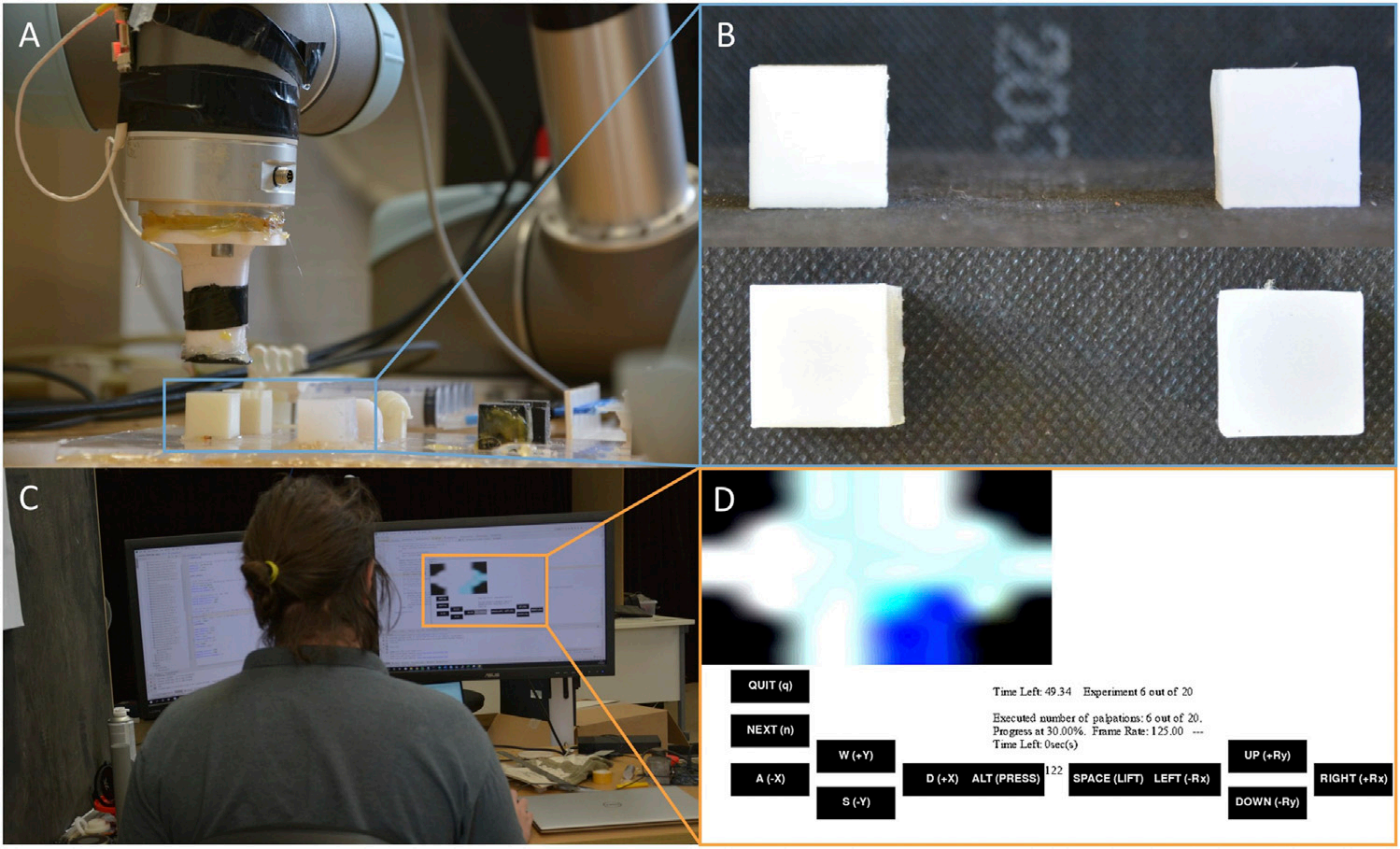
**(A)** The teleoperated robot with capacitive pressure sensor at the end-effector, **(B)** the lateral and top view of the two dummies used in the experiment, **(C)** the subject remotely controlling the robot with the keyboard, and **(D)** the interface showed by the leader with the visual map of haptic data.

To implement a remote operation setup, we use a 6 Degree of Freedom (DoF) UR5 Robotic Arm (Universal Robots) that can perform complex end-effector trajectories (see Figure 1).

The robot is placed in front of the workbench used to perform the experiments and it is directly controlled by a “server” workstation *via* TCP/IP. A remote “client” machine can then achieve teleoperation of the robot by connecting to the server through a graphical user interface (GUI) client program. The communication is implemented *ad hoc* for these experiments via sockets, and appropriate sensing delays from the server to the client can therefore be induced at will. The operator can access visual feedback corresponding to a pressure map detected by a distributed tactile sensor array placed at the tip of the end-effector. In the proposed study, we restrict the robot’s movements to vertical motions and translations. In every trial, the end-effector is placed at a random height, between 0.5 and 2.5 cm over either a soft or a hard dummy: the two dummies are both 8 cm^3^ cubes but the hard one is 3D printed with ABS, whereas the soft one is obtained by silicone casting Ecoflex 00-10 into a 3D printed mold. The starting height is randomized to avoid possible biases due to the adaptation of the subjects to the trials. Every subject is asked to remotely control the robot and to make contact with one of the dummies, also randomly selected in each trial, and to determine if the dummy is soft or hard. All subjects undergo a calibration trial in which they are made to control the robot to touch both dummies while knowing the correct answer. After the first trial, the subjects are then to perform the experiment 10 times for each condition. The different conditions include different introduced delays and different control modalities: in this work we tested delays of 0, 2 and 4 s jointly with four different control modalities (*HC*, *PHC*, *PRC* and *PHRC*), which will be described in Section 2.2.1, for a total of 120 trials for subject. The experiments have been performed block-wise, so all 10 trials for a given condition are completed before changing the condition itself. The conditions are tested in the following order: concerning the modalities, *HC* first, followed by *PHC*, *PRC* and *PHRC*, and for each modality the 0 s added delay has been tested first, followed by 2 and 4 s. A total of five subjects with no previous experience of remote task teleoperation or technology-assisted navigation have taken part in the experimental session, overall resulting in 600 trials. Among the participants, two subjects have a background in engineering and have previously interacted with robotic platforms, but not similar to the follower-leader architecture employed in this study. Given the length of the overall experiment (roughly 3 h) under the constant supervision of the authors, the study has been limited to five people.

**FIGURE 2:**
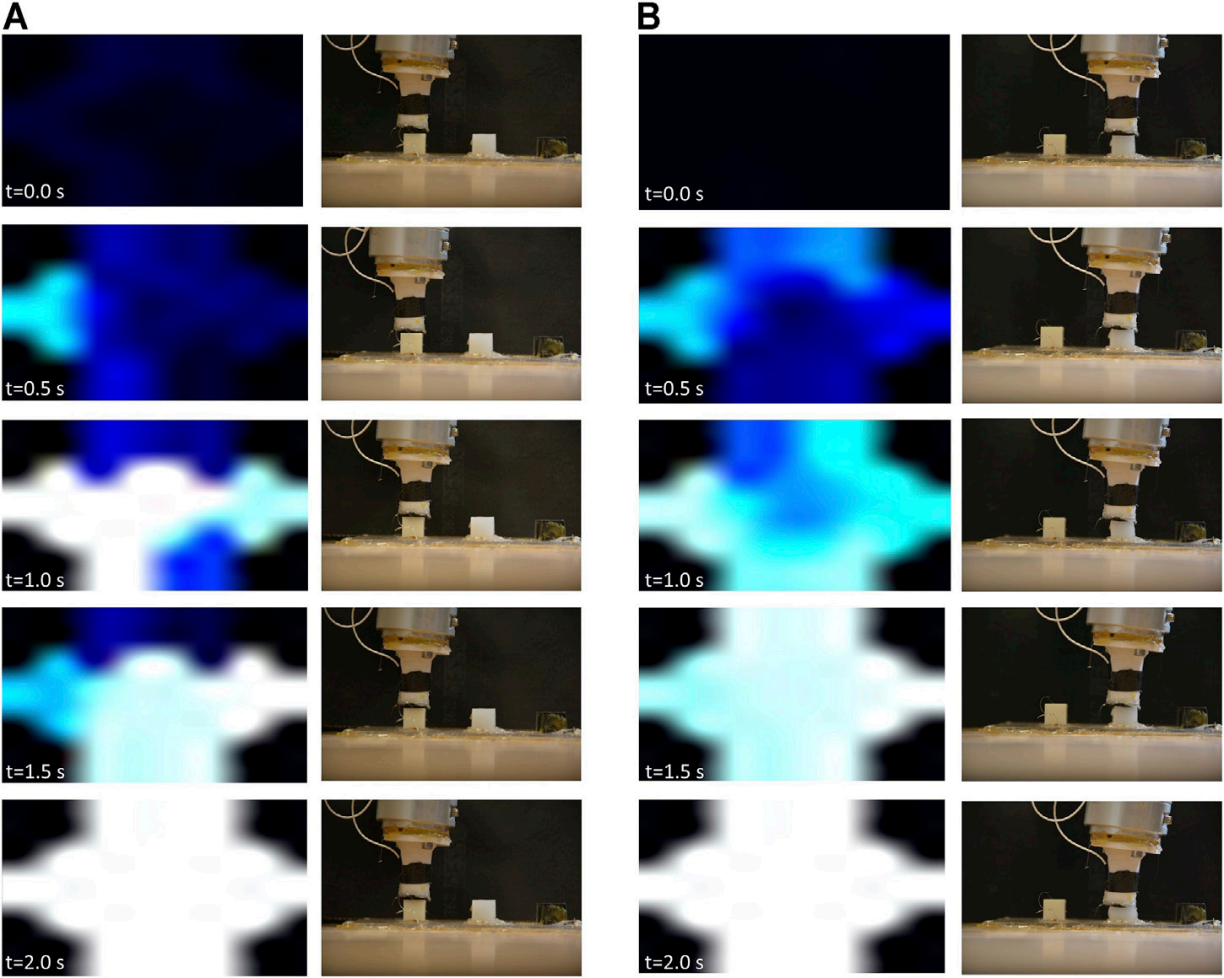
Robot states and corresponding sensor response upon contact with hard **(A)** and soft **(B)** dummies. The brighter areas (white) correspond to high recorded pressures by the corresponding taxels, while darker (blue and black) areas correspond to lower pressures. The brightness values are dynamic and change based on the history of touched objects on each trial, where they are normalized based on the highest and lowest recorded values by each taxel. In order to maintain the contact’s spatial information, the sensor data have been displayed according to the hexagonal geometry of the sensor, which is over-imposed onto a square black background.

The sensor used to collect haptic data is a hexagonal capacitive sensor array with seven tactile elements, or “taxels”, providing high sensitivity and spatial distribution over the surface of the sensor. The sensor provides measurement with a resolution of 16 bits corresponding to a variation of capacitance proportional to the pressure acting on top of the sensor. Details of the specific sensor and its fabrication have been previously reported (Scimeca et al., 2018; Maiolino et al., 2013). The collected data are normalized with respect to the maximum and minimum of each individual taxel and spatially calibrated to a visual representation of the pressure map showed to the user (see Figure 2).

### 2.2 Control

The control architecture is composed of a leader, a follower, and two communication channels: one from the leader to the follower and one from the follower to the leader. The implemented architecture falls in the category of unilateral teleoperation systems, due to the absence of a kinesthetic feedback: the haptic data are visualized as an haptic map in the GUI rather than used directly as haptic feedback. The leader sends to the follower the motor commands according to the selected control modality, and receives the delayed sensor’s information.

The predictive modalities of operation investigated assume the possibility of achieving an accurate predictive system of the tactile sensor response. As mentioned in Section 1, many solutions have been proposed for accurate prediction, including deep learning architectures, however, these are beyond the scope of this work. In these experiments, we assume near-perfect prediction of the robot position, given the user control inputs, under delayed conditions. We thus use a third, non-delayed, channel from the follower to the leader, which allows the sensor prediction system to observe near-real-time robot positions. This, in turn, allows us to observe more rigorously only the effects of sensor delay on the human response, and to dissociate the human response outcomes from the quality of the robot position prediction under delay conditions.

In the following sub-sections, we will first analyse the leader and its control modalities and later describe the follower and how it reacts to the motor commands.

#### 2.2.1 Leader

**FIGURE 3:**
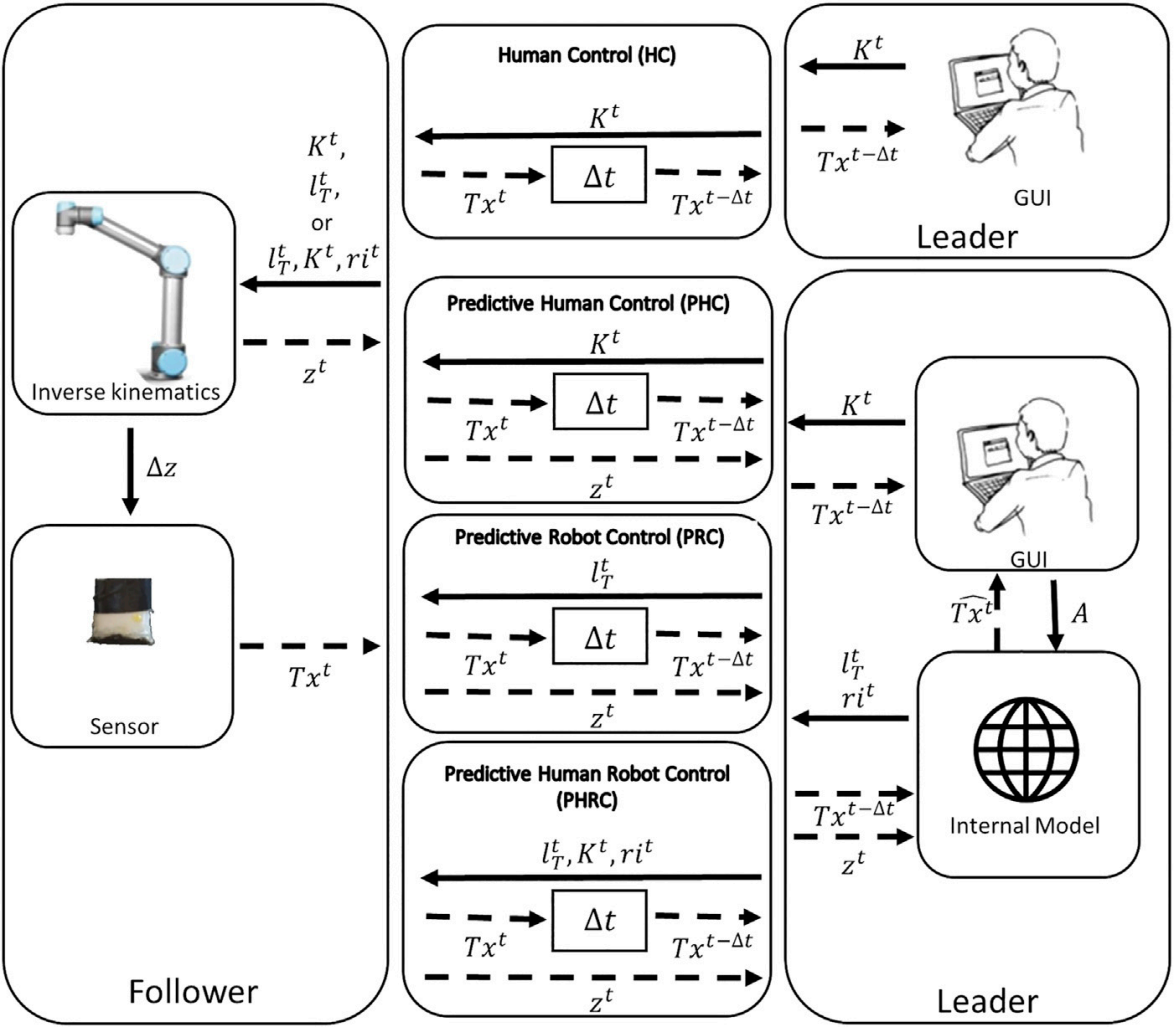
Schematics of the control in the four different modalities: *HC*, *PHC*, *PRC*, *PHRC*, from top to bottom. In the figure, *K* represents the information about the keys pressed by the user (“up” and “down”), *l*_*T*_ is the target position selected by the internal model, *ri* is the robot initiative, *A* is the answer given by the user at the end of the task, *z* is the information about the *z* coordinate of the end-effector, Δ*z* is the motion of the end-effector along the same direction, *Tx* is the sensor’s data about the taxels, and $\hat{Tx}$ is the predicted sensor’s data obtained from the internal model. The apex *t* is used for any not delayed signal whereas Δ*t* is the introduced delay.

The leader, located on the client machine, is composed of a graphical user interface (GUI) and an internal model. The GUI shows to the operator the haptic map and it is used to remotely move the robot. The leader can be configured in four different modalities: *HC*, *PHC*, *PHRC* and *PRC* (see Figure 3).

***HC***: In the Human-Control modality the response of the sensor *Tx* ^*t*^ is delayed of the desired amount Δ*t*, and the only motor commands sent to the follower are the keyboard’s inputs *K* ^*t*^. Since the motion is limited to the vertical axes, *K* ^*t*^ can contain the “up” and “down” keys, and it is empty when the operator is not touching the keyboard: an empty *K* ^*t*^ is interpreted as “stay”, thus stopping the robot.

***PHC***: In the Predictive-Human-Control modality we exploit the internal model to predict the real-time response. At each time step *t*, the model takes as inputs the delayed sensor’s information *Tx* ^*t*−Δ*t*^ and the delay-free height information *z* ^*t*^ and outputs the delay-free sensor’s prediction ${\hat{Tx}}^{t}$ , the target position $l_{T}^{t}$ and the robotic initiative *ri* ^*t*^. A detailed explanation of how the model itself is able to compute such variables will be provided later in this section. The *PHC* modality follows the same protocol as *HC*, with the exception that the predicted signal ${\hat{Tx}}^{t}$ is shown to the user in addition to the delayed signal *Tx* ^*t*−Δ*t*^.

***PRC***: In the Predictive-Robot-Control modality, the user input *K* ^*t*^ is ignored and the leader only sends the information about the target position $l_{T}^{t}$ . The user can still observe both the delayed and predictive sensor response *Tx* ^*t*−Δ*t*^ and ${\hat{Tx}}^{t}$ .

***PHRC***: In the Predictive-Human-Robot-Control modality, the leader sends commands *K* ^*t*^, $l_{T}^{t}$ and *ri* ^*t*^ to the follower, while still observing delayed and predictive sensor response *Tx* ^*t*−Δ*t*^ and ${\hat{Tx}}^{t}$ . Regardless of the control modality, at the end of each trial, the user is asked to select if the touched dummy was the hard or the soft one, and the next trial would automatically start after the choice. As already mentioned, subjects are asked to perform a block of 10 trials for each conditions’ combination, and the internal model continuously gathers data throughout the block and is reset only when the conditions are changed.

**FIGURE 4:**
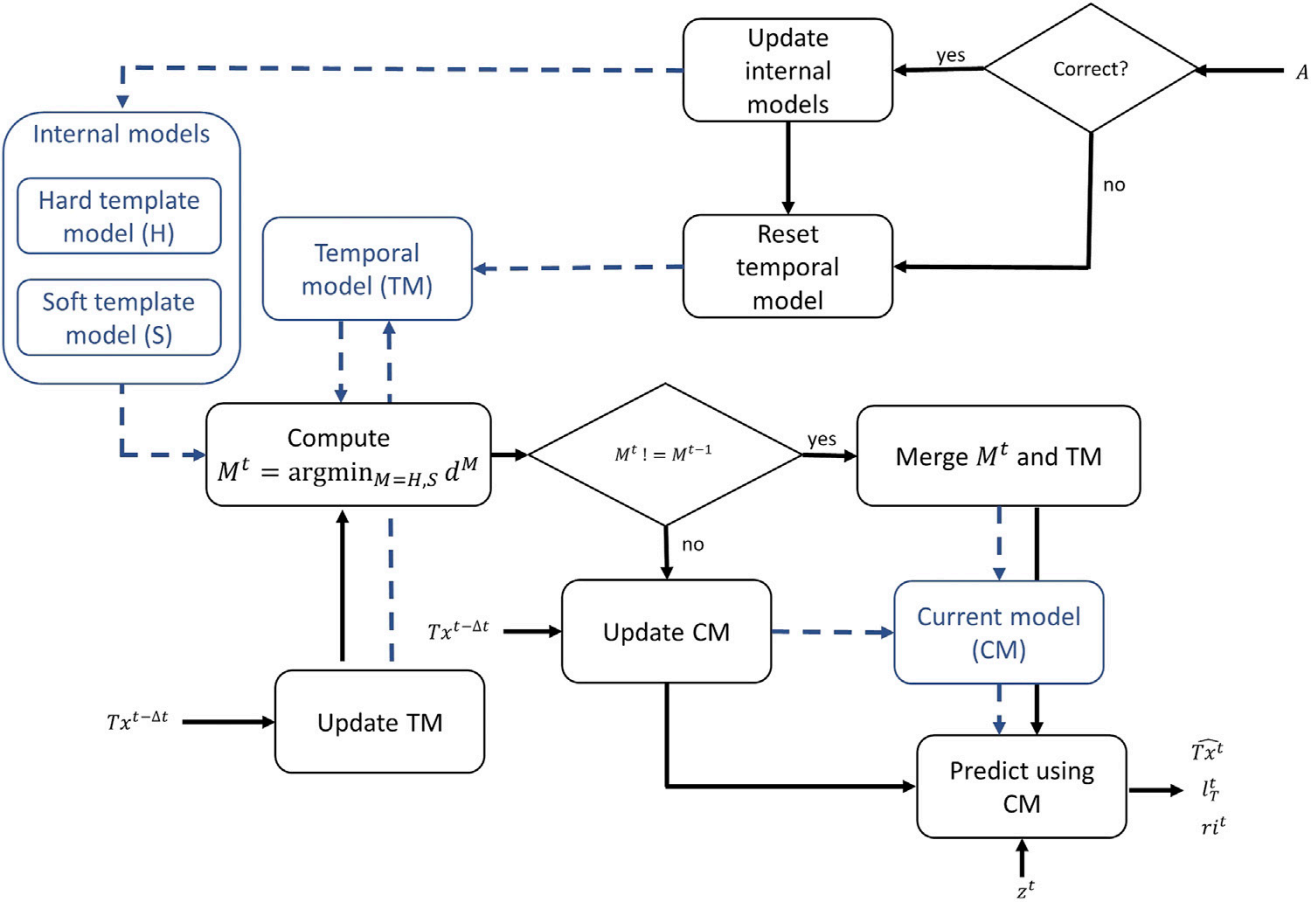
Internal model flow-chart: during the trial, the internal model continuously uses incoming data *Tx* ^*t*−Δ*t*^ to determine which model can better fit the collected data. The same data are then used to update both *TM* and *CM* according to Eq. 1. When the closest model (*M* ^*t*^) changes from *H* to *S* or *vice versa*, the *CM* is generated by merging *M* ^*t*^ and *TM* (see Eq. 4). The black lines correspond to the flow chart and the blue ones to data storage. The prediction of ${\hat{Tx}}^{t}$ and the values for $l_{T}^{t}$ and *ri* ^*t*^ is then given by showing the *CM* level corresponding to the height *z* ^*t*^. When the user gives the answer *A*, *TM* is reset and the internal models are updated if the given answer is correct.

The dummy used for the task is randomly selected at the beginning of each trial. Since the leader is unaware of the selected dummy’s identity, the internal model must capture several task environments. We have implemented four different sub-models: the hard and soft template models (*H* and *S*, respectively), the temporal model (*TM*), and the current model (*CM*). *H* and *S* represent the *a priori* information about the dummies known before the start of the trial by prior contacts with the object, whereas *TM* is reinitialized every time the operator provides an answer (*A*) and represents the *a posteriori* information derived from the ongoing trial. Finally, *CM* is the sub-model used for the prediction ${\hat{Tx}}^{t}$ (see Figure 4). Note that the model on the client-side updates its variables based on the sensory response from the server, and thus their updates are subject to delays (Δ*t*) should these be introduced. However, they hold the memory of previously observed sensor values and thus can be used to simulate the real-time sensory information for the operator. As a result of this implementation, we are able to have two different versions of the internal model, one for each dummy. This strategy can be scaled up to an arbitrary number of separate versions by adding template models.

Every sub-model is a Gaussian predictive model that divides the task space into *N* × *J* normally-distributed random variables, where *N* is the number of height levels (controlling the spatial resolution) and *J* is the number of taxels relevant for the haptic mapping. For the remainder of the experiments *N* = 50 and *J* = 7. For each random variable we compute a mean *μ*_*ij*_ and variance $\sigma_{ij}^{2}$ , where *i* ∈ *N* is the i^*th*^ height level and *j* ∈ *J* is the j^*th*^ taxel in the sensor array. Under the assumption of Gaussian distributed data, every new sensor data point $x_{ij}^{t}$ , received at time *t*, the mean and standard deviation of the corresponding random variables are updated as follows:

$$\left\{\begin{array}{cc} \mu_{ij}^{t}=\frac{\mu_{ij}^{t-1}N_{i}+x_{ij}^{t}}{N+1}\; & \forall \;x_{ij}^{t}\in Tx^{t-\Delta t}\\ {\sigma_{ij}^{t^{2}}}^{}=\frac{{\sigma_{ij}^{t-1^{2}}}^{}N_{i}+{\left(x_{ij}^{t}-\mu_{ij}^{t}\right)}^{2}}{N+1}\;\\ N_{i}=N_{i}+1\; & \text{if }N_{i}<N_{max} \end{array}\right.$$ (1)

where *N*_*i*_ is the number of samples collected for level *i* and *N*_*max*_ is the maximum number of points kept in memory, 10 in our case. At each iteration, the distance between *TM* and the models for the two dummies is computed as follows:

$$\left\{\begin{array}{cc} d_{i}^{M}=\overset{J}{\underset{j=1}{\sum }}{\left(\mu_{ij}^{M}-{\hat{\mu }}_{ij}\right)}^{2}\; & \text{if }{\hat{N}}_{i}>0\\ d_{i}^{M}=0\; & \text{if }{\hat{N}}_{i}=0 \end{array}\right.$$ (2)

$$d^{M}=\overset{N}{\underset{i=1}{\sum }}d_{i}^{M}$$ (3)

where the model *M* is either *H* or *S*, $d_{i}^{M}$ is the distance of a single level *i* and *d* ^*M*^ is the total distance between the two models, ${\hat{N}}_{i}$ is the number of samples for level *i* in *TM*, $\mu_{ij}^{M}$ is the mean value of taxel *j* and level *i* for the *a priori* model, soft or hard, and ${\hat{\mu }}_{ij}$ is the same value for *TM*. The model corresponding to the smallest distance is then selected and merged with *TM* as follows:

$$\left\{\begin{array}{c} \mu_{ij}^{W}=\frac{N_{max}-{\hat{N}}_{i}}{N_{max}}\mu_{ij}^{M}+\frac{{\hat{N}}_{i}}{N_{max}}{\hat{\mu }}_{ij}\;\\ {\sigma^{W}}_{ij}^{2}=\frac{N_{max}-{\hat{N}}_{i}}{N_{max}}{\sigma^{M}}_{ij}^{2}+\frac{{\hat{N}}_{i}}{N_{max}}{\hat{\sigma }}_{ij}^{2}\; \end{array}\right.$$ (4)

where *M* and *W* refer to the a priori template model selected and *CM*, respectively, and the *ˆ* denotes *TM* variables. For the sake of computational time, the merging only occurs in the first iteration and when and if the selected template model changes. In all the other iterations, *CM* is simply updated together with *TM*. When a new *CM* is created by merging, the old *CM* is deleted from the internal memory. When the user provides an answer, *TM* is reset and a new trial starts. Moreover, if the answer was correct, the corresponding model in the internal memory, *H* or *S*, is updated with the data of the last trial. *CM* is then used for the real time prediction ${\hat{Tx}}^{t}$ at the position *z* ^*t*^ and it is shown to the user together with the real delayed data *Tx* ^*t*−Δ*t*^.

In *PRC*, as we already discussed, *CM* is used by the robot to decide autonomously the best position in order for the operator to answer correctly, and the operator can only answer, but it is unable to move the robot. The target position $l_{T}^{t}$ is selected calculating the average mean value of every level of the model as follows:

$$\left\{\begin{array}{cc} l_{i}=\overset{J}{\underset{j=1}{\sum }}\mu_{ij}^{W}\; & \forall \;i\in 1,2\ldots n\\ l_{T}^{t}=\underset{l\in \left\{l_{1},l_{2}\ldots l_{n}\right\}}{\mathrm{arg min}}\left(l-\left(\frac{l_{max}+l_{min}}{2}\right)\right)\; \end{array}\right.$$ (5)

where *l*_*max*_ and *l*_*min*_ are the maximum and the minimum *l*_*i*_ among all levels, respectively. In *PRC*, the robot independently reaches the target position. The position is selected as the one corresponding to the average value of the sensor so to maximize the information shown to the operator and avoid the saturation of the sensor while making sure that the contact has happened.

In *PHRC*, the leader also computes the error (*e*) corresponding to each level and the robot initiative (*ri* ^*t*^) relative to the target position which will be used by the follower to combine the autonomous and human commands, as follows:

$$e_{i}^{t}=\frac{\sum_{j=1}^{J}{\sigma^{W}}_{ij}^{2}}{N_{i}^{W}}$$ (6)

$$\left\{\begin{array}{cc} ri^{t}=\frac{e_{T}^{max}-e_{T}^{t}}{e_{T}^{max}}\; & \text{if }N_{T}>1\\ ri^{t}=0\; & \text{if }N_{T}\leq 1 \end{array}\right.$$ (7)

where $e_{T}^{max}$ is the maximum value of *e* for the target position *T* and $e_{T}^{t}$ is the current error value for the same level.

#### 2.2.2 Follower

The follower, placed on the server workstation, is programmed to operate at a higher frequency than the leader, around 125 *Hz*. It receives the motor commands for the leader and executes them. In the *HC* and the *PHC* mode, the follower moves the end-effector at a speed of 0.5 *mm*/*s* in the direction specified by the corresponding key pressed by the operator. When operating in autonomous mode, the end-effector is driven toward the target position at the same speed, and in the case of shared control (*PHRC*), the selected speed is determined as follows:

$$v_{s}=ri^{t}v_{a}+\left(1-ri^{t}\right)v_{r}$$ (8)

where *v*_*a*_ and *v*_*r*_ are the desired autonomous and remote input speeds, and *v*_*s*_ is the resulting shared control speed. Note that *v*_*a*_ and *v*_*r*_, when not 0, are equal in magnitude but could be different in sign. The follower also communicates to the leader the sensor’s data needed to create the haptic map and saves all the robot’s data at the end of each trial.

## 3 Results

### 3.1 Control Modalities

**FIGURE 5:**
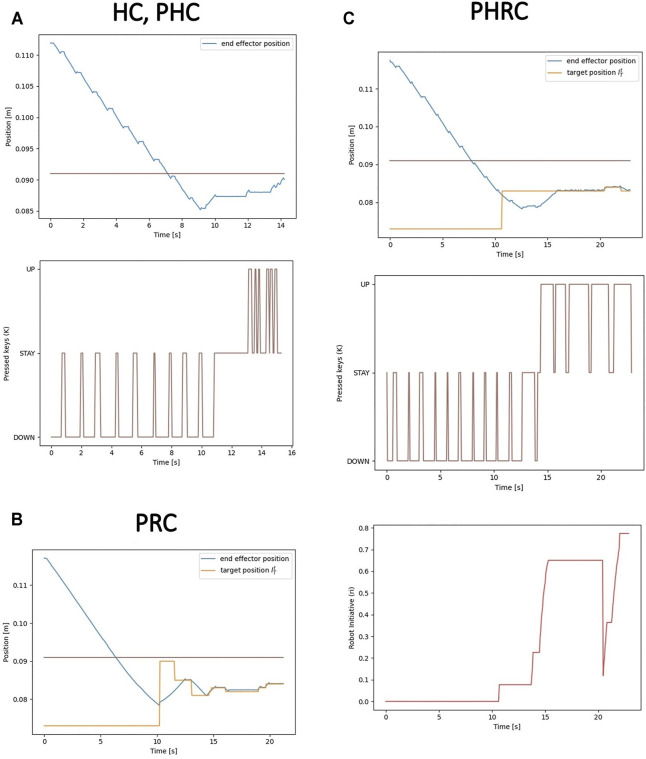
Examples of the different controlling modalities in 0 s delay condition. In *HC* and *PHC*, the motion is entirely controlled by the keyboard inputs given by the user **(A)**. In *PRC*, the input is completely ignored and the robot is driven toward the target position determined by the internal model **(B)**. In *PHRC*, both the target position and the input keys are used to determine the motor command given to the robot, according to Eq. 8 **(C)**. In the trajectories’ plots, the horizontal line is the height at which the top surface of the dummy is positioned.

As introduced in the previous section, all subjects undergo blocks of 10 trials for each delay level (0, 2 and 4 s) and each control modality. In all the modalities, the sensor data is delayed by a fixed and constant Δ*t*. We mainly want to study the effects of the magnitude of fixed delays, and as a result we do not consider time-varying delay conditions. The only difference between the 10 trials is the initial starting point of the end-effector, which is randomly generated in a range of 0.5–2.5 cm from the object: in order to avoid user’s adaptation to the task, the starting point is randomized, thus the users do not know the distance that they have to cover before entering in contact with the dummy’s surface. First, we analyse how the different modalities can control the trajectory of the end-effector. Figure 5 shows examples of how the three possible inputs can control the motion, all in a 0 s added delay condition, and how different control modalities result in different achieved trajectories. In the case of *HC* and *PHC*, the only control input are the pressed keys *K* ^*t*^. As it can be seen in sub-figure (A), that creates a jerky trajectory because the robot stops every time the user is not pressing any key, given that $l_{T}^{t}$ is ignored for these modalities. Since the starting height is unknown, the subject tends to stop every few seconds in every delay condition to avoid sudden impact. Therefore, the subject is able to stop soon after the contact with the object, and answer usually within 20 s. Conversely, *PRC* shows exactly the opposite trends: the trajectories are a lot smoother, but the end-effector reaches a much lower position before the internal model is able to process enough data in order to correctly update the model change the target position $l_{T}^{t}$ . In this modality, *K* ^*t*^ is ignored and not taken into consideration. Finally, in *PHRC*, both inputs are accounted, as explained in Eq. 8. The magnitude of *ri* depends on how much data are gathered about $l_{T}^{t}$ , therefore it increases as the end-effector spends time in $l_{T}^{t}$ . At the same time, the data gathered by the sensor can shift the $l_{T}^{t}$ , resetting *ri* to a new value. It can be noticed that the trajectory is initially very similar to the sub-figure (A) because *ri* is close to 0. Then, as *ri* increases, the trajectory rapidly converges to the target position, with little oscillations. When the $l_{T}^{t}$ changes, around 20 s, *ri* drops, but it rapidly recovers as soon as enough data are collected from the new target.

### 3.2 Delay Introduction

**FIGURE 6:**
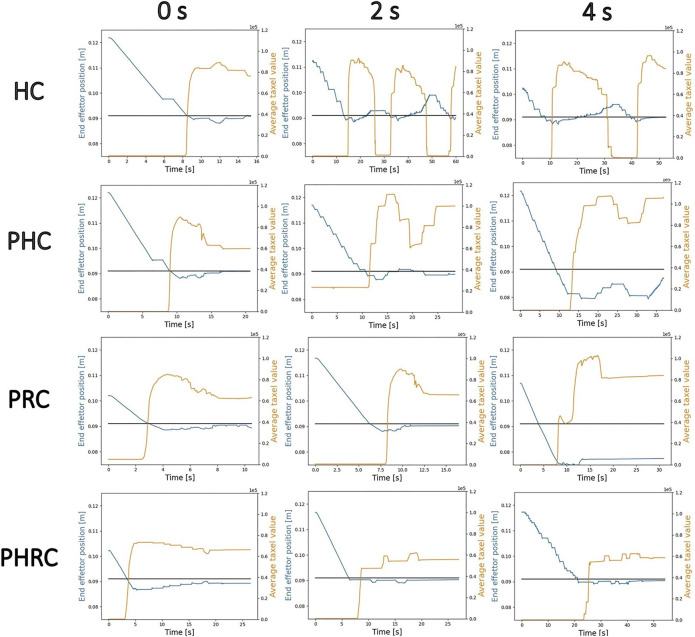
Examples of trials for different delays and all four different control modalities: each row represent a control modality and each column represent a delay condition. In blue the *z* position of the end-effector and in orange the sum of the taxels’ value. The black line indicates the position of the dummy’s surface.

Next, we are interested in how the introduced delay affects the different modalities, so we introduce a delay of 2 and 4 s to the communication channel from follower to leader. Figure 6 shows some trials taken from the experimental data. In all the graphs, it is shown the trajectory of the end-effector of the robot and a proxy of the delayed feedback showed to the user, obtained by performing the sum of all taxels’ values present in *Tx* ^*t*−Δ*t*^. The sum of the taxels has been selected as a metric because it summarizes effectively all the haptic information seen by the user. At 0 *s* delay all four modalities have similar behaviors: the end-effector stops when the contact is detected and then it undergoes small adjustments to reach the correct height at which is possible to recognize the touched object without the sensor’s saturation nor detachment.

In the case of *HC*, adding delay causes the formation of “chattering”: the users are able to see that they have touched too late, therefore they do not stop the end-effector in time and they saturate the sensor’s readings. Then, trying to move back to the correct height, they are once again unable to stop at the correct height and the end-effector moves away from the object, causing the sensor’s signal to drop back to 0. Chattering is an example of indirect instability, since it is originated by the user overcompensating the perceived errors due to the delay added to the visual representation of the haptic map. Compared with the 2 s delay case, the 4 s delay has longer and deeper oscillations, and the elapsed time before the answer is very high in both cases when compared with the 0 s case. Concerning the *PHC*, we can still observe some oscillations, but their magnitude is strongly reduced: it is evident that the users are able to use the information about the predicted output to minimizing overshooting, thus reducing chattering and giving an answer faster.

Next, the *PRC* shows almost no chattering because it does not rely on the user’s input, but the delay directly affects the update timing of $l_{T}^{t}$ : both in the 2 and 4 s case the target position is only updated after 2 and 4 s after the previous one is reached, respectively. This could cause potentially dangerous contacts if the end-effector and the object exchange high forces for a prolonged time: this is evident in the 4 s delay case, in which we can see the end-effector almost “vibrating” at its lowest point, indicating that it was trying to go further but it was stopped by the environment. We also know that such a contact saturated the sensor’s signal because $l_{T}^{t}$ is changed as a consequence of it. Moreover, it can also be noticed that this modality does not allow any oscillation, once the final $l_{T}^{t}$ is determined: in all the remotely controlled modalities with no delay the users use a small oscillation as a strategy to successfully complete the task. However, this modality showcases much faster trials’ time, likely due to the absence of chattering. Finally, in *PHRC* we can still observe a little oscillation, but it is mixed with the target following behavior proper of *PRC*. This modality is shown to be slightly slower than *PRC*, but it allows to some extent small movements and deviations from the target position, thus ensuring to some extent the user’s freedom of motion.

**FIGURE 7:**
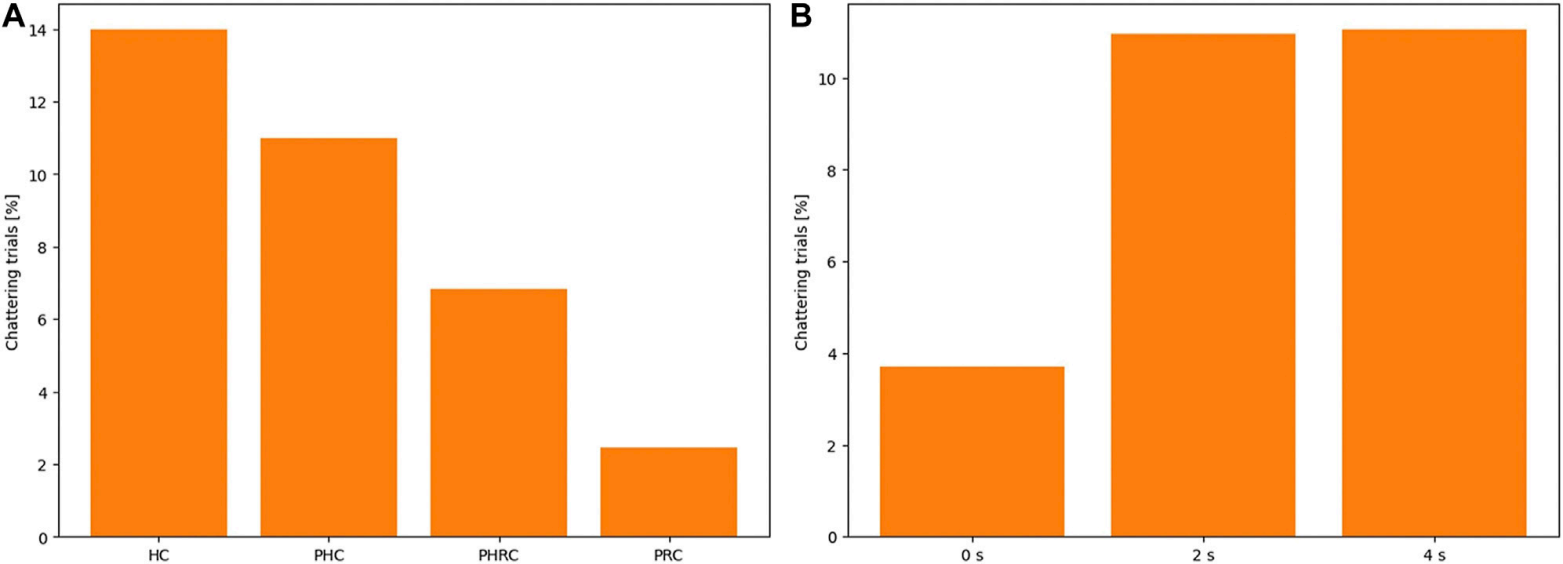
Total percentages of chattering trials among all subjects and all conditions, for a total of 600 trials, as a function of the different modality **(A)** and the delay condition **(B)**. Chattering is defined as the detachment of the sensor from the dummy’s surface after initial contact is made.

To further validate our hypothesis, we have analyzed the percentage of cases in which we can detect chattering, defining chattering as the presence of at least one detachment from the dummy after initial contact is made. Figure 7 shows the cumulative percentage computed among all trials and all subjects. It is clear that chattering is common in remotely control modalities, especially *HC*, but can be strongly reduced by introducing autonomy. Note that the modalities are listed from the least autonomous, *HC*, on the left, to the most autonomous, *PRC*, on the right. Therefore, autonomy in the system can be considered a valid answer to strongly limit such behavior. To the right, it can be noticed that introducing any delay strongly increases the chattering, but going from 2 to 4 s does not produce additional chattering: as already discussed, this further increase in delay only produces greater and wider oscillations, not affecting the presence of chattering, but its severity.

### 3.3 Group Study Results

**FIGURE 8:**
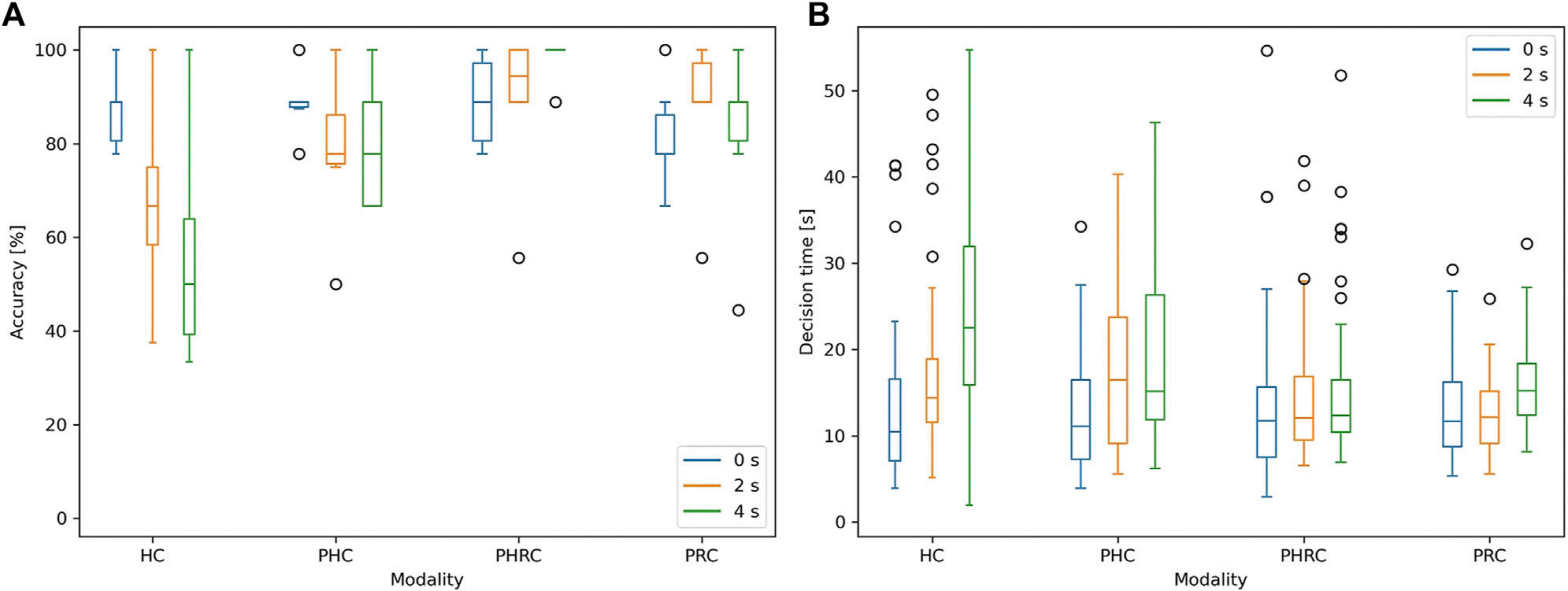
Box plots of accuracy **(A)** and decision time **(B)** as a function of the delay introduced, in the four different control modalities. Decision time is the time elapsed from the first contact to the subject’s answer.

The main results shown in Figure 8 are the accuracy, to the left, and the decision time, to the right. The decision time is calculated from the first contact made with the dummy to the moment in which the operator gives the answer, in order to avoid any bias related to the random initial height. The accuracy plot illustrates that there is a clear cut between *HC* and all the other modalities as the introduced delay increases: at 0 s delay, *HC*’s accuracy is 90*%*, but it rapidly decreases when the delay is introduced. The *PHC* also decreases in performance as the introduced delay increases, but less severely. Since both the fully remotely operated modalities have noticeably decreasing trends as the delay increases, we can infer that the delay compromises the stability of the feedback loop relying on the human action. On the other hand, the other two modalities seem not to be affected by delay as much, especially in the case of *PRC*, in which the accuracy stays between 80 and 85%. The *PHRC* seems to increase its performance as the delay increases: on one side, it could be inferred that *PHRC* is better exploited with delay, on the other side this may be due either to the small sample size or to the fact that every subject undergoes 0, 2 and 4 s delay trials in this specific order, thus they may find the control modality not intuitive at first, but learn and become better over time. We believe that *PHRC* is able to achieve better results than *PRC* because the user is able to slightly tune the motion of the end effector, thus exploring the contact zone and gather more useful information. Similarly, the decision time of all four modalities is within a 3 s difference when looking at the no-delay condition, but there is a clear difference among them as the delay increases. In high delay conditions, the four modalities are largely separated: *HC* is the slowest, followed by *PHC* and *PHRC*, and the fastest is *PRC*. We believe that this is due to the fact that both *PHRC* and *PRC* are effective in keeping the end-effector at the right range so as to avoid sensor saturation and contact detachment, thus not wasting time in regions with no information. Overall, *HC* and *PHC* are more sensitive and they lose stability when the delay is introduced, whereas *PRC* and *PHRC* show a much more robust behavior, showing that complete or partial automation is able to contrast the unstable behavior of delayed human-in-the-loop teleoperation.

**FIGURE 9:**
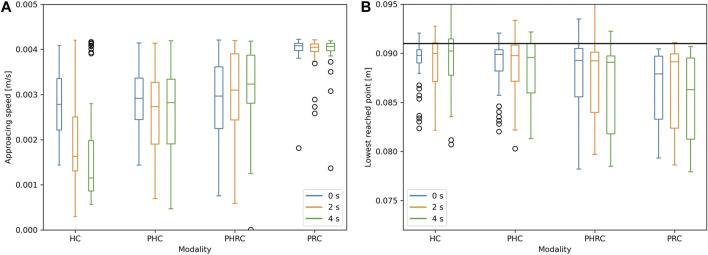
Box plots of approaching speed **(A)** and deepest reached point **(B)** as a function of the delay introduced, in the four different control modalities. In the deepest reached point's graph, the black line represents the height at which the first contact with the dummy is made.

Next, Figure 9 shows some more metrics useful to assess the performance: the deepest point reached during contact and the total average trial time. The deepest reached point is a proxy for how strong the interaction with the environment has been during the trial. Ideally, it is not needed to reach deep in the dummy in order to achieve good discrimination, and going too deep can produce high forces that could damage the robot or the environment. The plot illustrates that only the shared control mode seems to be affected by the delay added to the system. The autonomous mode always reaches deeper than the other modalities and the two fully remote-controlled modes show the best results, having a light interaction in all cases. Even when the delay is introduced, the subjects are able to avoid deep contacts by moving slower, thus they do not reach deeper than necessary in the dummies, but, as previously shown, they compromise the task execution’s speed. Shared control approaches the autonomous values as the delay increases, likely because the control architecture tends to trust the robot more than the remotely given motor commands. Concerning the approaching speed, the fastest modality is *PRC* and it is completely not affected by the delay: once the target position is set, the end-effector will approach the phantom at a constant speed *v*_*a*_, regardless of the sensor’s data. The other three modalities are quite close to each other for the 0 s case, but as the delay increases it is shown that *PHRC* tends to go toward the *PRC*, *PHC* decreases slightly and then stabilizes, and HC tends to decrease its performance. Once again, *PHRC* approaches *PRC* as the delay increases, likely due to the increased trust toward the internal model. While working in high delay conditions, if the user is left in charge of the motion, the motion will result jerky and oscillating, thus collecting data points for more height levels than a smooth trajectory: this additional data can make the internal model much more precise, thus increasing *ri* ^*t*^ and consequently approaching the trajectories of the *PRC*.

**FIGURE 10:**
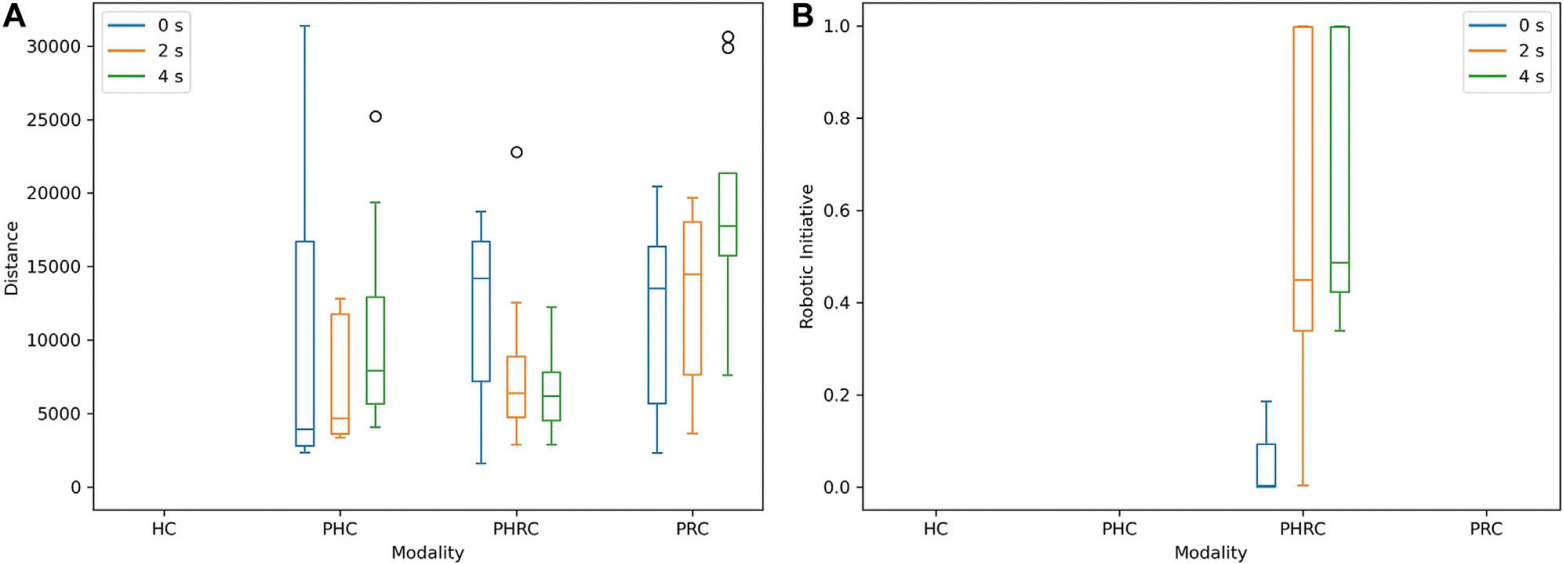
Box plots of average distance between predicted output ${\hat{Tx}}^{t}$ and real output *Tx* ^*t*^ **(A)**, and average robotic initiative *ri* **(B)**. Average *ri* is computed by using Eq. 8 and taking the mean value for 0 ≤ *t* ≤ *t*_*end*_.

More in detail, Figure 10 illustrates the precision of the internal model in all the different conditions. The distance between prediction and real data is calculated as follows:

$$D=\frac{\int_{\;0}^{t_{end}}\sum_{j=1}^{J}{\left({\hat{x}}_{j}^{t}-x_{j}^{t}\right)}^{2}dt}{t_{end}}\;\forall \;x_{j}^{t}\in Tx^{t}\wedge {\hat{x}}_{j}^{t}\in {\hat{Tx}}^{t}$$ (9)

where *t*_*end*_ is the overall duration of the trial. The data show that the internal model is able to give a better prediction in *PHRC* than in the other modalities, especially as the introduced delay increases. The increasing trend of *ri* with respect to delay further supports that the introduction of delay makes the model more reliable and thus makes the robot behaving more autonomously (see Eq. 8). Moreover, both too little and too much autonomy seem to result in lower performance upon the introduction of delay. For *PHC*, the prediction’s accuracy decreases as the delay increases because the model is trying to predict the sensor’s output for wider and more chattering trajectories, whereas, in *PRC*, it decreases because the sensor gathers data from a lower number of different levels. Compared with *PHC*, *PHRC* allows voluntary small oscillations that can gather data from adjacent levels, increasing the overall quality of the model, and thus the average value of *ri*.

**FIGURE 11:**
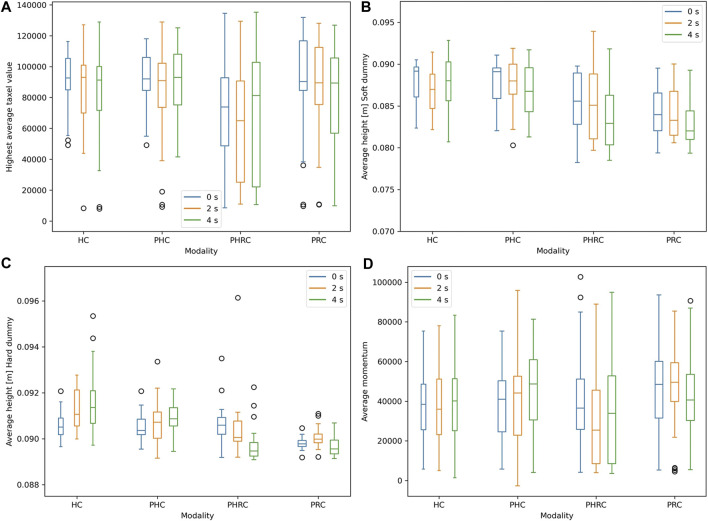
On top, average height after contact in the case of hard **(A)** and soft **(B)** dummies. On the bottom, the maximum recorded taxels’ value **(C)** and the trials’ average momentum **(D)**, both used to quantify the strength of the interaction between robot and environment.

To further study how the interaction differs among soft and hard dummy, the average height while interacting with the environment and the interaction forces are reported in Figure 11. Due to the different stiffness of the two dummies, the end-effector reaches deeper when interacting with the soft dummy in all cases. When comparing trials belonging to the same dummy, it is evident that during *PRC* the subjects tend to interact more with the surface of the dummy, reaching deeper. Concerning the soft dummy, it is also clear that the magnitude of the delay affects the performance, likely due to the slower updating speed of the internal model. When analyzing the interaction forces, we focused on the maximum registered taxels’ value and the average momentum. For each trial, the average momentum is computed using the average taxels’ value to calculate the impulse of the trial, later averaged using the total time of each experiment, as follows:

$$M=\frac{\int_{\;0}^{t_{end}}\sum_{j=1}^{J}x_{j}^{t}dt}{t_{end}}\;\forall \;x_{j}^{t}\in Tx^{t}$$ (10)

Both metrics show that *PHRC* is able to achieve on average softer interactions, exchanging less force with the environment and thus achieving a safer performance. Even if the interaction force is not considered as a main result, it is preferable to maintain it as low as possible, as long as it does not negatively affect the main performance metrics.

The high variance shown is mostly due to inter-subject, as opposed to intra-subject, variability. This is explained by both the starting baseline and the general ability to recognize the two different objects, which largely varies from person to person.

## 4 Discussion

This work investigates humans’ behavior in teleoperation as communication delay increases and proposes a possible solution to minimize its effects on performance: an internal model to predict future sensory data, and shared control. In scientific literature, multiple studies tried to predict future sensors signal using either statistical methods or NN, but we introduce shared control as an additional tool on top of a statistical predictor. Shared control, especially when used in a repetitive task, has already been proven to enhance precision and task speed, and we show that it is also an effective strategy against possible errors due to communication delay. More in detail, this study compares four different control modalities at increasing degree of autonomy (DoA): *non-predictive human control (HC)*, *predictive human control (PHC)*, *(shared) predictive human-robot control (PHRC)* and *predictive robot control (PRC)*. Every modality has been tested with 0, 2, and 4 s delays added to the communication channel from follower to leader. The experimental protocol is composed of blocks of 10 trials for every combination of modality and delay, for a total of 120 trials, and has been carried out on five subjects with no previous experience of remote task teleoperation or technology-assisted navigation.

Each block needs to be completed before moving to the next one and the internal model is reset between blocks. The proposed unilateral teleoperated system is composed of a leader and a follower: the leader is composed by a GUI shown on the user’s computer screen, that displays the haptic map received from the follower and allows the user to move the end-effector with the keyboard, and an internal model, based on a hierarchical arrangement of Gaussian sub-models, used to predict delay-free signals, whereas the follower is made of a UR5 robotic arm, that executes the leader’s imposed motions, and a capacitive pressure sensor, used to gather data for the haptic map.

The results clearly show that the *HC*, which is the standard control modality for state-of-the-art teleoperation, is extremely sensitive to added delay: the subjects tend to oscillate more during the trials and the overall performance results less accurate and slower both in approaching the phantom and taking a guess after the contact is made. Just adding a prediction of the sensor’s signal, in *PHC*, is enough to strongly limit the oscillations and to noticeably increase the accuracy and approaching speed, but the decrease in decision time is not substantial. In both cases, the delay causes indirect instabilities through the user’s overcompensation and creates strong deviations from the intended trajectories. The shared control architecture, *PHRC*, and the autonomous one, *PRC*, show faster timings and increased performance, likely due to the increase autonomy of those modes, that allows them to avoid indirect instabilities and maintain a good contact with the dummies, without detachment or sensor’s saturation. All in all, both modalities showcase a stable behavior even in high delay conditions, thus we consider the addition of some DoA to be a stabilizing factor for delayed teleoperation. Nevertheless, the *PHRC* shows slightly higher accuracy than *PRC* as the delay increases: by observing the 0 s delay cases it can be noticed that small oscillations are often used as a strategy during the task, and those voluntary small oscillations can still be performed during *PHRC*, but not during *PRC*, given the complete autonomy of this modality. Moreover, it can also be noticed that the *PRC* reaches deeper than the other three modalities, regardless of the operating conditions, and this produces high contact forces that could harm the environment or the sensor: ideally, we want to be able to recognize the touched object without strong physical interaction with the object itself. Finally, from the results, it can be observed that *PHRC*’s performance tends to approach *PRC*’s as the delay increases, and this is explained by the shared control trusting more the internal model in high delay conditions: high delays produce high oscillations, and this also means that more data from different height levels are collected faster, thus making the model more reliable and increasing the autonomy of this modality. In other words, when the user performs wider trajectories, it gathers data from a wider range, thus increasing rapidly the internal model’s precision, thus leading the system toward a more autonomous behavior, and those types of trajectories are affected by the introduced delay: higher delay produces longer and greater oscillations. One limitation within the experimental conditions lies within the long trials needed to gather the relevant data from each participant. This in turn has limited the number of participants to 5. However, whilst harder to capture the cross-participant variance of the results, the findings show a coherent trend for each individual across several trials in different conditions, supporting the results shown in this work.

We showed that both signal prediction and shared control can be used to minimize the negative effects of delay in teleoperation, up to 4 s, especially concerning indirect instabilities. Overall, the *PHRC* is proven to be a good trade-off between fully user-driven modalities and complete autonomy, having high accuracy, fast decision time, average approaching speed, and moderate contact interaction. The choice of the experimental scenario in this paper was limited to a 1 DoF reaching and recognition task. In more challenging tasks, the system necessary to achieve appropriate haptic interactions can have several DOFs, inducing longer training times by the user as well as higher degrees of control skills. Additional work on tasks with higher degrees of system control could shed some light onto whether the complexity of the task (or the controller) can also influence the ability of shared control to mitigate communication delays. We analyzed the problem of communication delay from follower to leader, but the delay is bidirectional in real-life scenarios, and this issue is not considered in our discussion. Moreover, we focused on the effect of delay’s magnitude, thus adopting constant delays during the experiments. While this prevents the generalization of the results to time-varying delay for now, future work could extend this study to such a case, under the assumption that the predictive system is capable of capturing the varying delayed sensor response. As a closing remark, if shared control can be used to minimize or nullify the effect of communication delay, it could make teleoperation possible over long distances and unstable internet connections, such as teleoperating a robot through space or performing a medical examination on a patient located in an unreachable location.

## Data Availability

The original contributions presented in the study are included in the article/Supplementary Material, further inquiries can be directed to the corresponding author.

## References

[B1] Abdelaal A. E. Mathur P. Salcudean S. E. (2020). Robotics *In Vivo*: A Perspective on Human-Robot Interaction in Surgical Robotics. Annu. Rev. Control. Robot. Auton. Syst. 3, 221–242. 10.1146/annurev-control-091219-013437

[B2] Ajoudani A. Zanchettin A. M. Ivaldi S. Albu-Schäffer A. Kosuge K. Khatib O. (2018). Progress and Prospects of the Human-Robot Collaboration. Auton. Robot 42, 957–975. 10.1007/s10514-017-9677-2

[B3] Anderson R. J. Spong M. W. (1989). Bilateral Control of Teleoperators with Time Delay. IEEE Trans. Automat. Contr. 34, 494–501. 10.1109/9.24201

[B4] Attanasio A. Scaglioni B. De Momi E. Fiorini P. Valdastri P. (2021). Autonomy in Surgical Robotics. Annu. Rev. Control. Robot. Auton. Syst. 4, 651–679. 10.1146/annurev-control-062420-090543

[B5] Avgousti S. Christoforou E. G. Panayides A. S. Voskarides S. Novales C. Nouaille L. (2016). Medical Telerobotic Systems: Current Status and Future Trends. Biomed. Eng. Online 15, 1–44. 10.1186/s12938-016-0217-7 PMC4983067 27520552

[B6] Buvik A. Bugge E. Knutsen G. Småbrekke A. Wilsgaard T. (2016). Quality of Care for Remote Orthopaedic Consultations Using Telemedicine: A Randomised Controlled Trial. BMC Health Serv. Res. 16, 483–511. 10.1186/s12913-016-1717-7 27608768 PMC5017045

[B7] Farahmandrad M. Ganjefar S. Talebi H. A. Bayati M. (2020). A Novel Cooperative Teleoperation Framework for Nonlinear Time-Delayed Single-Master/multi-Slave System. Robotica 38, 475–492. 10.1017/S0263574719000791

[B8] Farajiparvar P. Ying H. Pandya A. (2020). A Brief Survey of Telerobotic Time Delay Mitigation. Front. Robot. AI 7, 578805. 10.3389/frobt.2020.578805 33501338 PMC7805850

[B9] Ferrell W. R. (2013). Remote Manipulation with Transmission Delay. IEEE Trans. Hum. Factors Electron. HFE 6, 24–32. 10.1109/thfe.1965.6591253

[B10] Ficuciello F. Tamburrini G. Arezzo A. Villani L. Siciliano B. (2019). Autonomy in Surgical Robots and its Meaningful Human Control. Paladyn 10, 30–43. 10.1515/pjbr-2019-0002

[B11] Hauser K. (2013). Recognition, Prediction, and Planning for Assisted Teleoperation of Freeform Tasks. Auton. Robot 35, 241–254. 10.1007/s10514-013-9350-3

[B12] Huang J.-Q. Lewis F. L. Liu K. (2000). Neural Net Predictive Control for Telerobots with Time Delay. J. Intell. Robotic Syst. Theor. Appl. 29, 1–25. 10.1023/A:1008158209668

[B13] Huang L. Yamada H. Ni T. Li Y. (2017). A Master-Slave Control Method with Gravity Compensation for a Hydraulic Teleoperation Construction Robot. Adv. Mech. Eng. 9, 168781401770970. 10.1177/1687814017709701

[B14] Inoue S. Toyoda K. Kobayashi Y. Fujie M. G. (2009). “Autonomous Avoidance Based on Motion Delay of Master-Slave Surgical Robot,” in Proceedings of the 31st Annual International Conference of the IEEE Engineering in Medicine and Biology Society: Engineering the Future of Biomedicine, EMBC 2009, Sepember 2-6, 2009, Minneapolis, MN, 5080–5083. 10.1109/IEMBS.2009.5333458 19964112

[B15] Ivanova E. Carboni G. Eden J. Kruger J. Burdet E. (2020). For Motion Assistance Humans Prefer to Rely on a Robot Rather Than on an Unpredictable Human. IEEE Open J. Eng. Med. Biol. 1, 133–139. 10.1109/ojemb.2020.2987885 PMC8974791 35402952

[B16] Javdani S. Admoni H. Pellegrinelli S. Srinivasa S. S. Bagnell J. A. (2018). Shared Autonomy via Hindsight Optimization for Teleoperation and Teaming. Int. J. Robot. Res. 37, 717–742. 10.1177/0278364918776060

[B17] Kanaishi K. Ishibashi Y. Huang P. Tateiwa Y. (2020). “Effect of QoS Control for Cooperative Work between Remote Robot Systems with Force Feedback,” in Proceedings of the 4th International Conference on Advanced Information Technologies, ICAIT 2020, Sepember 28-30, 2020, Taoyuan, Taiwan, 94–98. 10.1109/ICAIT51105.2020.9261798

[B18] Liu Y. C. (2015). Robust Synchronisation of Networked Lagrangian Systems and its Applications to Multi‐robot Teleoperation. IET Control. Theor. Appl. 9, 129–139. 10.1049/iet-cta.2013.0914

[B19] Luo J. He W. Yang C. (2020). Combined Perception, Control, and Learning for Teleoperation: Key Technologies, Applications, and Challenges. Cogn. Comput. Syst. 2, 33–43. 10.1049/ccs.2020.0005

[B20] Maiolino P. Maggiali M. Cannata G. Metta G. Natale L. (2013). A Flexible and Robust Large Scale Capacitive Tactile System for Robots. IEEE Sensors J. 13, 3910–3917. 10.1109/JSEN.2013.2258149

[B21] Mehrdad S. Liu F. Pham M. T. Lelevé A. Atashzar S. F. (2021). Review of Advanced Medical Telerobots. Appl. Sci. 11, 209–47. 10.3390/app11010209

[B22] Moustris G. P. Hiridis S. C. Deliparaschos K. M. Konstantinidis K. M. (2011). Evolution of Autonomous and Semi-autonomous Robotic Surgical Systems: A Review of the Literature. Int. J. Med. Robot. Comput. Assist. Surg. 7, 375–392. 10.1002/rcs.408 21815238

[B23] Muelling K. Venkatraman A. Valois J.-S. Downey J. E. Weiss J. Javdani S. (2017). Autonomy Infused Teleoperation with Application to Brain Computer Interface Controlled Manipulation. Auton. Robot 41, 1401–1422. 10.1007/s10514-017-9622-4

[B24] Nikpour M. Yazdankhoo B. Beigzadeh B. Meghdari A. (2020). Adaptive Online Prediction of Operator Position in Teleoperation with Unknown Time-Varying Delay: Simulation and Experiments. Neural Comput. Appl. 33, 1–18. 10.1007/s00521-020-05502-5

[B25] Okamura A. M. (2004). Methods for Haptic Feedback in Teleoperated Robot‐assisted Surgery. Ind. Robot 31, 499–508. 10.1108/01439910410566362 PMC1317565 16429611

[B26] Park J. Khatib O. (2006). A Haptic Teleoperation Approach Based on Contact Force Control. Int. J. Robot. Res. 25, 575–591. 10.1177/0278364906065385

[B27] Reddy S. Dragan A. Levine S. (2018). Shared Autonomy via Deep Reinforcement Learning. Robotics: Science and System (RSS), June 26-30, 2018, Pittsburgh, PA. 10.15607/rss.2018.xiv.005

[B28] Schultz C. Gaurav S. Monfort M. Zhang L. Ziebart B. D. (2017). “Goal-predictive Robotic Teleoperation from Noisy Sensors,” in Proceedings - IEEE International Conference on Robotics and Automation, May 29-June 3, 2017, Singapore, 5377–5383. 10.1109/icra.2017.7989633

[B29] Scimeca L. Maiolino P. Iida F. (2018). “Soft Morphological Processing of Tactile Stimuli for Autonomous Category Formation,” in 2018 IEEE International Conference on Soft Robotics, RoboSoft 2018, April 24-28, 2018, Livorno, Italy, 356–361. 10.1109/ROBOSOFT.2018.8404945

[B30] Sharifi M. Salarieh H. Behzadipour S. Tavakoli M. (2017). Stable Nonlinear Trilateral Impedance Control for Dual-User Haptic Teleoperation Systems with Communication Delays. J. Dynamic Syst. Meas. Control Trans. ASME 139, 121012. 10.1115/1.4037125

[B31] Sirouspour S. (2005). Modeling and Control of Cooperative Teleoperation Systems. IEEE Trans. Robot. 21, 1220–1225. 10.1109/TRO.2005.852254

[B32] Smith C. Jensfelt P. (2010). A Predictor for Operator Input for Time-Delayed Teleoperation. Mechatronics 20, 778–786. 10.1016/j.mechatronics.2010.03.002

[B33] Takagi A. Li Y. Burdet E. (2021). Flexible Assimilation of Human's Target for Versatile Human-Robot Physical Interaction. IEEE Trans. Haptics 14, 421–431. 10.1109/TOH.2020.3039725 33226954

[B34] Tanwani A. K. Calinon S. (2017). “A Generative Model for Intention Recognition and Manipulation Assistance in Teleoperation,” in IEEE International Conference on Intelligent Robots and Systems, Sepember 24-28, 2017, Vancouver, BC, 43–50. 10.1109/IROS.2017.8202136

[B35] Tao X. Li J. Huang Q. (2020). “Stability Analysis of Time-Varying Delay Bilateral Teleoperation System with State Prediction,” in 2020 6th International Conference on Control, Automation and Robotics (ICCAR), July 15-17, 2021, 2021, Dalian, China, (IEEE), 272–276. 10.1109/iccar49639.2020.9108050

[B36] Van Den Berg D. Glans R. De Koning D. Kuipers F. A. Lugtenburg J. Polachan K. (2017). Challenges in Haptic Communications over the Tactile Internet. IEEE Access 5, 23502–23518. 10.1109/ACCESS.2017.2764181

